# Turning a normal microscope into a super-resolution instrument using a scanning microlens array

**DOI:** 10.1038/s41598-017-19039-6

**Published:** 2018-01-12

**Authors:** Gergely Huszka, Martin A. M. Gijs

**Affiliations:** Laboratory of Microsystems, École Polytechnique Fédérale de Lausanne, 1015 Lausanne, Switzerland

## Abstract

We report dielectric microsphere array-based optical super-resolution microscopy. A dielectric microsphere that is placed on a sample is known to generate a virtual image with resolution better than the optical diffraction limit. However, a limitation of such type of super-resolution microscopy is the restricted field-of-view, essentially limited to the central area of the microsphere-generated image. We overcame this limitation by scanning a micro-fabricated array of ordered microspheres over the sample using a customized algorithm that moved step-by-step a motorized stage, meanwhile the microscope-mounted camera was taking pictures at every step. Finally, we stitched together the extracted central parts of the virtual images that showed super-resolution into a mosaic image. We demonstrated 130 nm lateral resolution (~λ/4) and 5 × 10^5^ µm^2^ scanned surface area using a two by one array of barium titanate glass microspheres in oil-immersion environment. Our findings may serve as a basis for widespread applications of affordable optical super-resolution microscopy.

## Introduction

The discovery of the photonic nanojet phenomenon generated by a lens-like dielectric micro-object opened a new chapter in optical microscopy in 2004^1^. Placing such micro-object over a sample allowed imaging with a resolution better than predicted by Abbe’s law of diffraction^2^. Since then, many groups investigated, the nature of the photonic nanojet phenomenon^3–8^. Recently, it became an accepted statement that the photonic nanojet is a narrow light beam with high optical density emerging over a length ~2λ away from the micro-object that created it with a full-width-at-half-maximum (FWHM) of ~λ/3^9^. Parallel to the investigations of understanding and imaging the phenomenon itself, different micro-objects that are capable of creating such a nanojet for imaging purposes were studied^10,11^, the most common of which being nano-scale lenses^2,12^, polymer microdroplets^13^ and dielectric spheres both in the nanometer^14^ and micrometer range^15,16^. Subsequently, the research focus shifted towards applications^17–26^, but in these papers super-resolution was achieved typically over a very small area that was comparable to the size of the used micro-object. Recently, it was demonstrated that the super-resolved area can be extended by various scanning methods^27–31^, including the super-resolution imaging ability of dielectric microspheres that were used in an atomic force microscopy (AFM) setup^32,33^. This technique extended the field-of-view of the imaging system, but also carried the drawbacks of an AFM system, namely the extreme sensitivity on vibration, requiring a dedicated setup. We have engineered the scanning principle and the super-resolution imaging capability of an array of dielectric microspheres into a robust experimental setup and thereby could upgrade a normal optical microscope to a super-resolution one.

## Experimental setup

**Figure 1:**
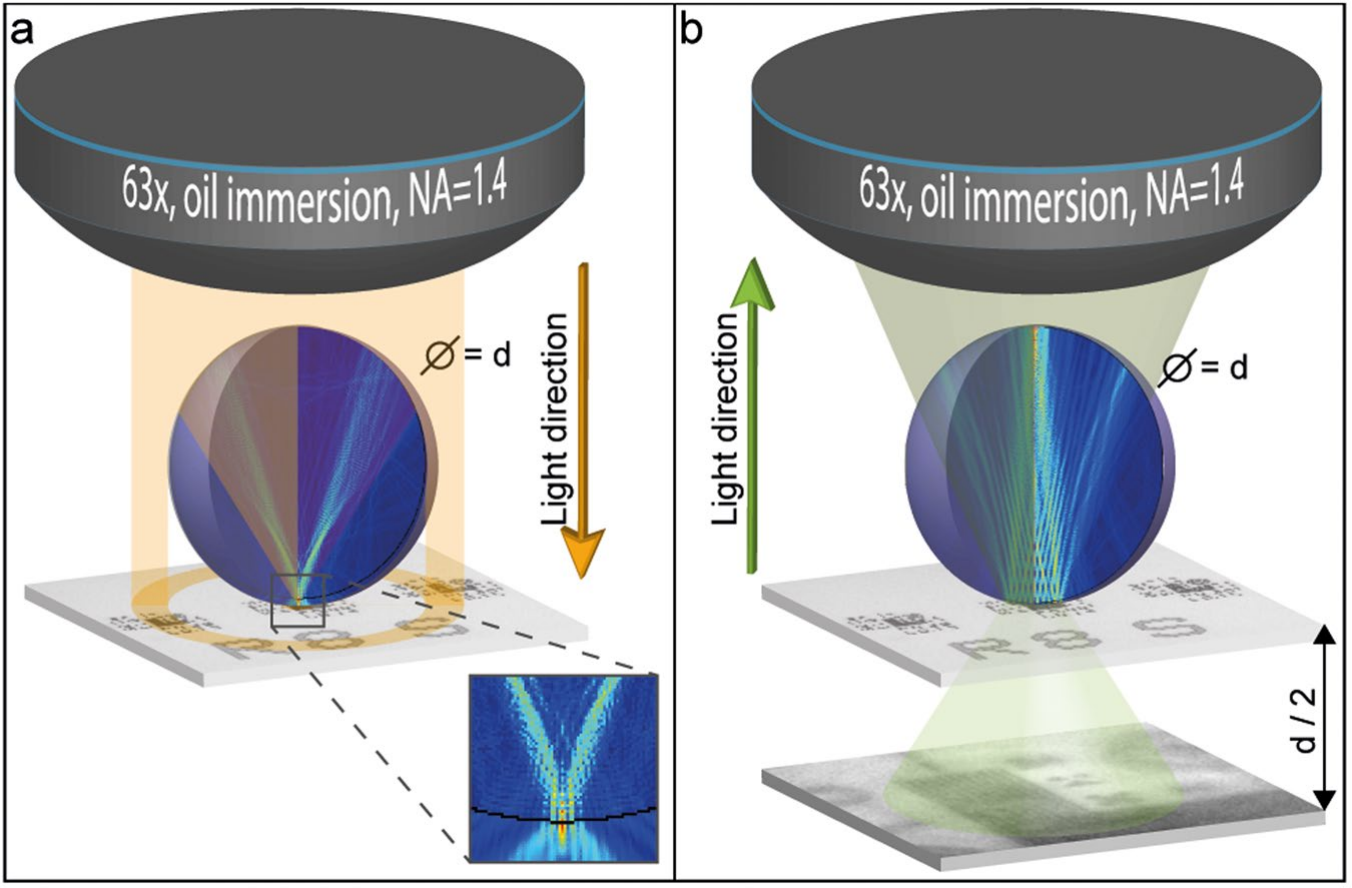
Operation principle of the imaging system. (**a**) Excitation: light approaches through the microscope objective towards the dielectric microsphere with diameter d. In absence of an object to be imaged in the light path, the dielectric microsphere generates a photonic nanojet on its shadow side, as is shown on the finite element simulation of the electric field in the inset. (**b**) If an object is present underneath the microsphere, reflection occurs: the simulation shows reflection from a sample consisting of a modulated pattern of eleven lines and spaces with dimensions below the diffraction limit. The modulation is preserved and the near-field information of the diffraction-limited sample is propagated into the far-field within the microsphere. At the same time, the microsphere acts as a lens and generates a virtual image at d/2 distance below the sample plane, as illustrated by the green cone.

The working principle of our imaging system is explained in Fig. 1. As shown in Fig. 1a, if a dielectric microsphere with refractive index *n*_*sphere*_ is placed underneath a light microscope’s objective and is surrounded by a medium with refractive index *n*_*medium*_, a photonic nanojet is created right under the microsphere. The position, shape and size of this photonic nanojet is determined by the wavelength of the illumination light (visible light in our study, 400 nm < *λ* < 700 nm, with peak at *λ* = 600 nm), the shape of the micro-object (which is spherical in our case) and the ratio between the two refractive indices (*n*_*sphere*_ = 1.95 for the used barium titanate glass – BTG – microspheres and *n*_*medium*_ = 1.56 for immersion oil). These parameters must be tuned to generate a photonic nanojet exiting exactly at the surface of the microsphere, to enable imaging sub-diffraction limited features of the sample^34,35^. When a sample is placed just underneath the microsphere and the incident light is reflected back from the sample (Fig. 1b) the modulation pattern from the sample is transferred through the microsphere, towards the microscope objective. However, besides development of the photonic nanojet, also the near-field interaction with the sample placed underneath the microsphere matters, so that the microsphere-based imaging resolution may become sample-dependent^36^. Therefore, in certain cases the super-resolution capability of a system can be only slightly better than the diffraction limit. Because of the geometrical optics properties of the microsphere, which acts as a lens, a virtual image will be projected about half the microsphere diameter distance below the sample plane. This virtual image plane can be placed in the focus of the microscope objective. An image, recorded while observing this plane will contain information about the sub-diffraction features, therefore enables super-resolution microscopy. The major drawback of such an imaging is that the field-of-view is limited to size of the central part of the microsphere. To overcome this limitation we established a scanning mechanism, with which we could restore the field-of-view to the full size of the microscope objective.

**Figure 2:**
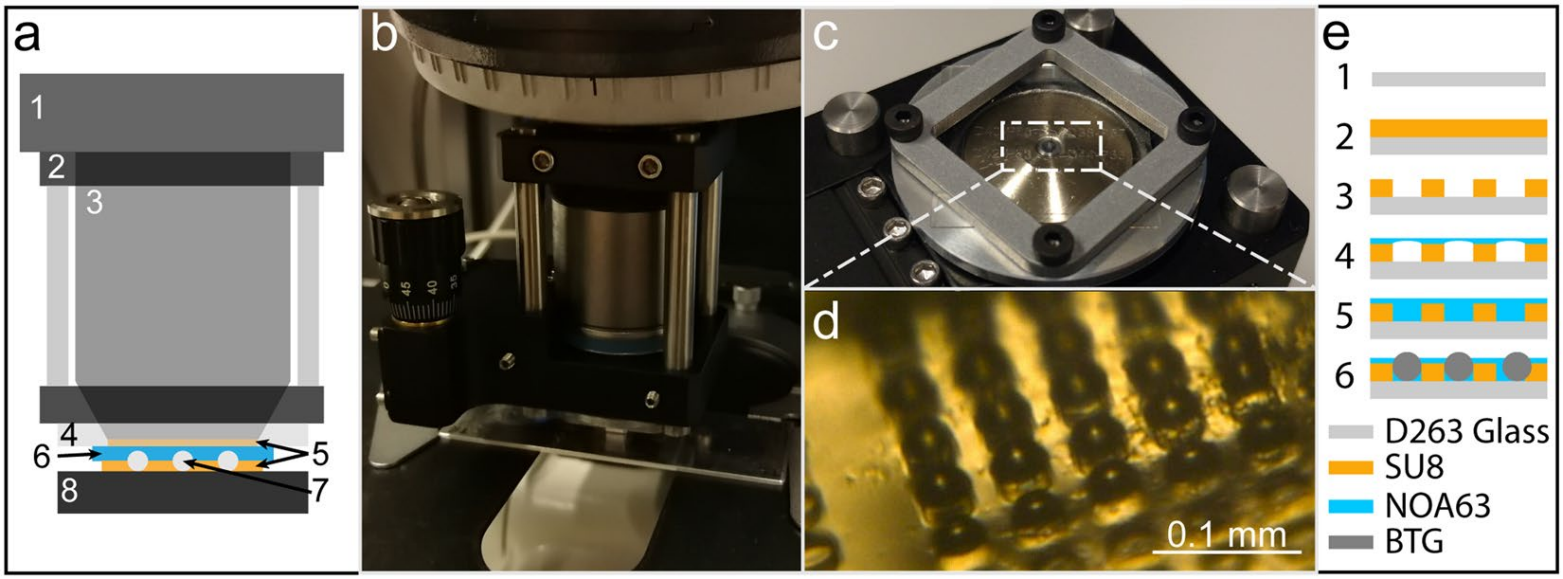
Imaging setup. (**a**) Schematic of the optical microscope with our imaging setup attached. 1: Microscope revolver. 2: Optical cage (black) with spacers (metallic rods). 3: Microscope objective. 4: Aluminum adapter. 5: Immersion oil. 6: Chip template. 7: BTG microsphere. 8: Sample to be imaged. (**b**) Photograph of the optical cage system and the Z-axis translator. (**c**) Photograph of the bottom part of the optical cage with a custom aluminum holder attached to the Z-axis translator that clamps a glass microsphere array chip. (**d**) Photograph of the fabricated microsphere array chip, showing that the microspheres emerge from the surface plane of the chip. (**e**) Schematic of the fabrication process of the microsphere array chip. 1: A cover glass chip is treated with oxygen plasma. 2: 20 µm SU8 photoresist is spin-coated on the chip. 3: An array of microwells is patterned into the SU8 with photolithography. 4: A layer of NOA63 optical glue is placed on the top, leading to air bubbles stuck into the microwells. 5: The air bubbles are removed with vacuum treatment. 6: BTG microspheres are patterned into the microwells and fixed by UV curing of the optical glue.

Our setup consists of two major components as shown in Fig. 2. The first is a metal frame, which is composed from 30 mm cage system parts (Thorlabs, Germany) including an SM1Z, Z-axis translator that are fixed to the microscope objective (Fig. 2a,b). An in-house designed and fabricated aluminum element is attached to the inside thread of the SM1Z translator (Fig. 2c), the aim of which is to fix a glass-based microsphere array chip onto the objective (Fig. 2d). The role of the Z-axis translator between the objective revolver and the chip holder is to enable focus adjustment along the Z-axis, as needed for positioning the chip in the right focal plane prior to imaging based on our previous study in this topic^37^. The array chip was fabricated in the clean room using negative photoresist-based photolithography (Fig. 2e). The dimensions of the chip substrate is 22 × 22 × 0.15 mm^3^ and it is made of D 263 M borosilicate glass (Menzel-Gläser, Germany). After oxygen plasma cleaning, it was coated with 20 µm 3025 type SU8 (MicroChem, USA). The glass-chromium mask used for the lithography consisted of an array of 40 µm diameter wells with a pitch of 60 µm. After development, a 4 µl droplet of Norland Optical Adhesive 63 (NOA63, Norland Products, USA) was spread on the top of the well array. Then the chip was placed in a vacuum chamber for 20 minutes to remove the air bubbles stuck in the wells of the SU8 layer. Subsequently, we placed 38–45 µm diameter BTG (Cospheric, USA) microspheres on the NOA63 layer and swiped them over the surface until they were located in the wells. The excess amount of microspheres was removed to prevent them acting as a spacer during imaging. Finally, the chip was exposed to UV light until an accumulated dose of 4.5 Joules/cm^2^ was reached, which is required for curing the NOA63 glue.

We placed our sample on a motorized microscope stage (Axio Imager M2m with HAL100 halogen light source, Zeiss, Germany) which was controlled by our custom algorithm. The scanning protocol was established as follows: after an initial focus setting along the Z-axis, the microscope-attached camera (AxioCam MRm, Zeiss, Germany) took a picture, when focused on the virtual image plane of the sample. To make a single scanning step, the stage moved 5 µm downwards along the Z-axis to prevent scratching the sample and took one step along either the X- or the Y-axis, where the in-plane step-size was set by the user before the scanning. Finally, it moved back to the original Z-axis position and was ready for taking the next picture. This scanning process was repeated until the pre-set sample area was fully scanned. Hereafter, the saved pictures were cropped to the region of interest (ROI) and subsequently stitched together to create a big field-of-view, super-resolution image. We implemented a stitching algorithm that overlapped the regions in the image that were just outside the ROIs, to keep the useful amount of super-resolution pixels at maximum. To achieve that, we used the fact that the scanning went along a predefined path and that the useful area of a taken photograph was always at the same position, so that its size could be calculated in advance. Because of this, we did not have to use the conventional stitching algorithms where the edges of the tiles are compared pixel-to-pixel for stitching. During experiments we used a 63×, oil immersion, NA = 1.4 objective, which limited the field-of-view to a 2 × 2 array of microspheres. Therefore, we had up to four ROIs per picture. Since each ROI was limited by the central part of the microsphere, we could not use conventional stitching algorithms.

## Results and Discussion

**Figure 3:**
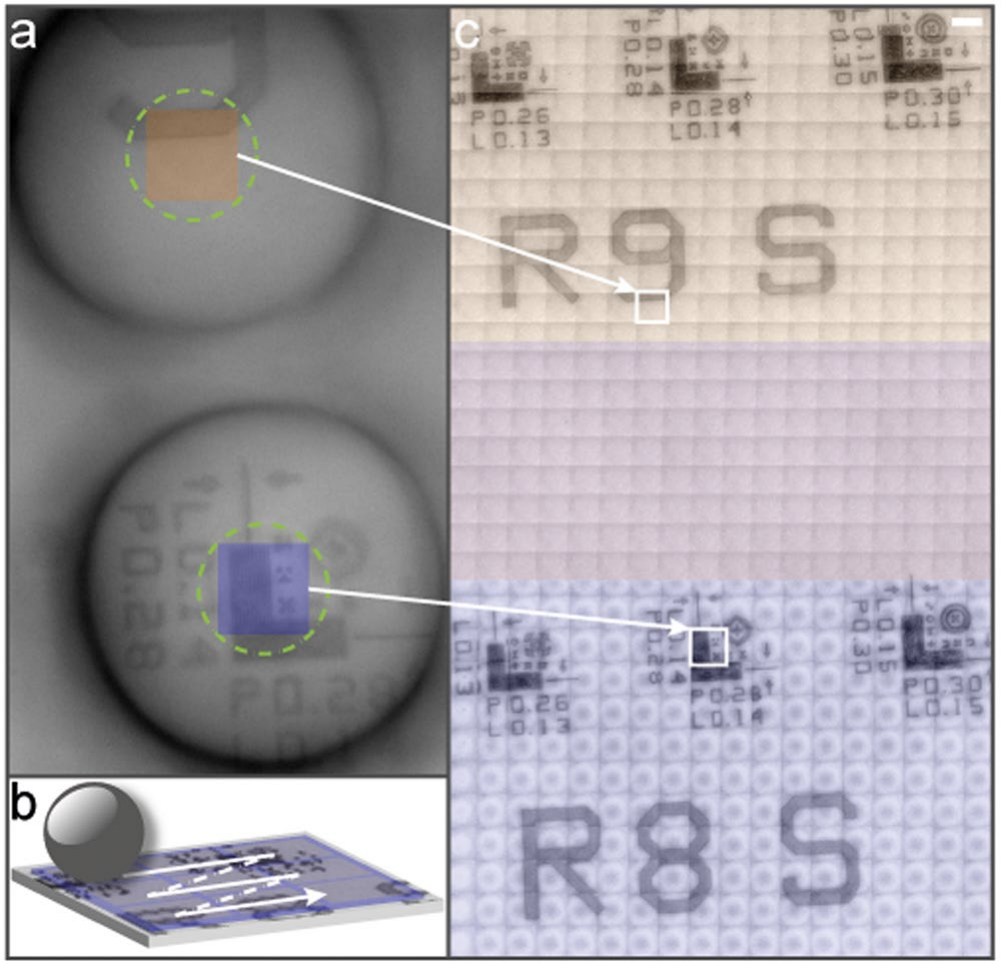
Demonstration of the microsphere scanning process. (**a**) Two microspheres of the array that are in the field-of-view of the microscope-mounted camera generate virtual images of the sample. The central regions of the microsphere-generated images (marked with green circles) show super-resolution. At every step of the scanning process, the inside squares (marked with yellow for the first and with blue for the second microsphere, respectively) are retained for generating the final image. (**b**) Schematics of the step-by-step scanning that is carried out using a motorized stage, controlled by an in-house developed scanning algorithm. (**c**) Final image at the end of the process. First, individual tiles, two of which are indicated by the white squares, are extracted from the center of the microsphere images and are stitched together to form a mosaic image. Next, the thus-generated mosaic images of the individual microspheres, indicated by yellow and blue tiles, are combined. Since the pitch of the microsphere array is smaller than the scanned area, overlap between the yellow tiles from the first microsphere and blue tiles from the second microsphere occurs. Scale bar 5 µm.

In Fig. 3a, one can see a typical image captured from the virtual image plane. Technically, up to four microspheres could fit into the field-of-view of the camera. Practically, because of the size distribution of the microspheres and the dependence of the sensitivity of the detection principle on the local distance between the sample and the microsphere surface, we chose to use two microspheres for easy simultaneous imaging. In the center of the two microspheres (marked with the green dashed circles in Fig. 3a) super-resolution imaging is enabled. The yellow and the blue rectangles mark the ROI that will be extracted for the final image. During imaging, the microspheres have a fixed position on the pictures, while the sample is scanned (Fig. 3b). In Fig. 3c a composed image of a silicon-based microscope calibration target (MetroBoost, USA) is shown. The calibration target shows L-shaped line-space patterns with 130, 140 and 150 nm line width, from the left to right, respectively. The patterns are repeated in every row; therefore, the patterns in row nine (marked as R9 S) are nominally the same as the ones in row eight (marked as R8 S). One can observe the individual tiles that were used for stitching (yellow and blue corresponds to the two microspheres) and the overlap between the two scanned areas. The reason for this overlap is the pre-set scanning parameters, as the step-size was set to 5 µm along both X- and Y-axis, meanwhile the full scanned area was 100 × 100 µm. Since the pitch distance of the microspheres is 60 µm, this resulted in a 40 µm-wide overlap area. Based on these results, it is possible to see the two major advantages of implementing scanning with multiple microspheres. With such a configuration, the scanning time could be reduced or the imaged area could be increased. The gain is proportional to the number of microspheres used during the process in both cases.

**Figure 4:**
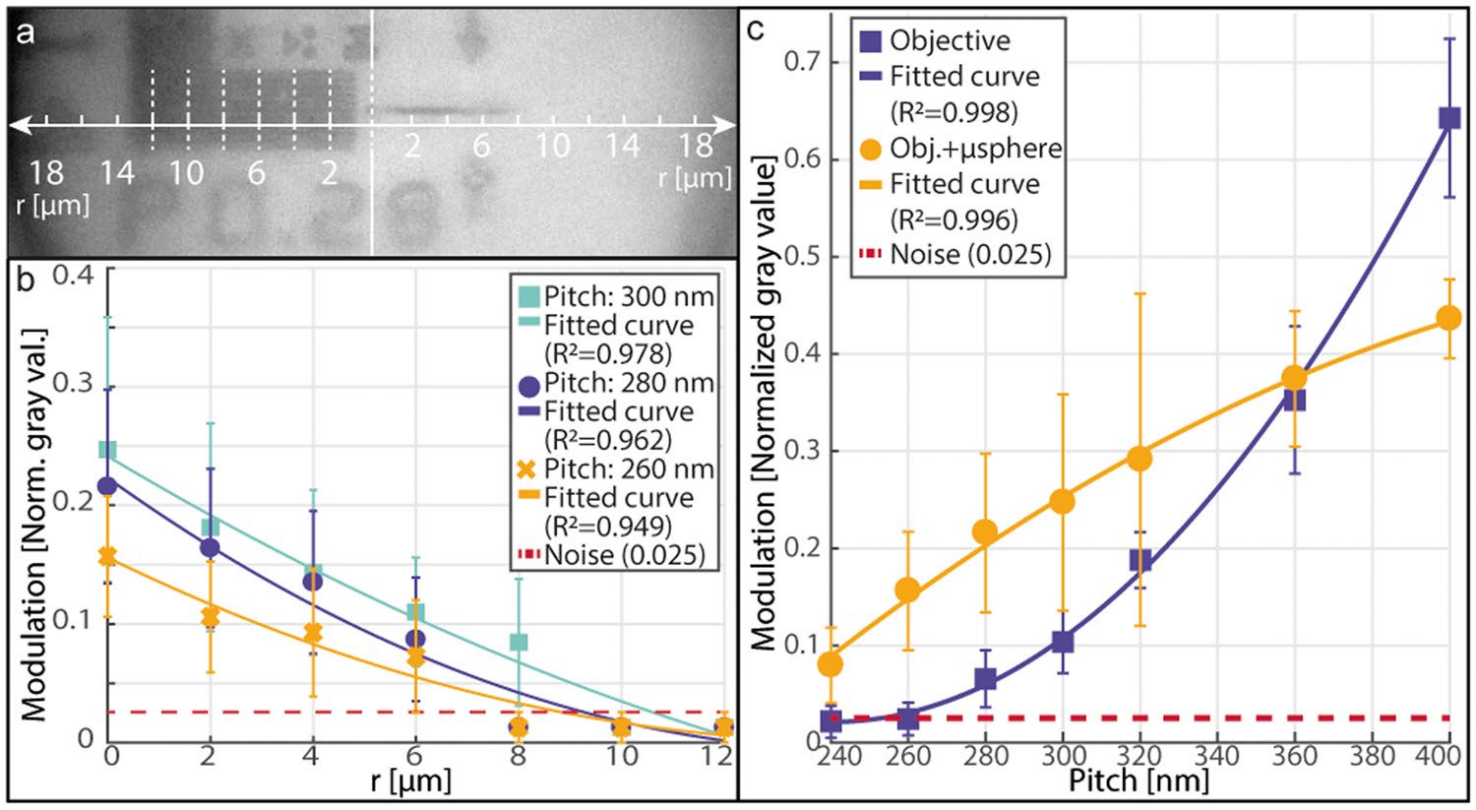
Analysis of the modulation of the imaged line-space patterns. (**a**) Micrograph of an eleven-line 140 nm-wide line-space pattern, as imaged by a microsphere, showing that the modulation pattern is best resolved towards the center of the microsphere and is attenuated with increasing radial distance r. (**b**) Measurement of the modulation as a function of r in the case of 260 nm, 280 nm and 300 nm pitch, respectively, along the dashed lines indicated in (**a**). (**c**) Measurement of the modulation of differently sized line-space patterns obtained at r = 0. Circles show the measured values when the microsphere array chip was present, while squares show the performance of the microscope objective without the array chip. Points are averages over the eleven lines at a given r, error bars: ±SD.

To determine the imaging performance of our system, we measured the modulation of line-space patterns with different lateral dimensions as shown in Fig. 4. Figure 4a is a typical example of an image of 140 nm line-space pattern, showing that lines are better resolved towards the center of the microsphere and less sharp image is generated for increasing radial distance *r*. Experimentally we placed a 524–565 nm band-pass filter (AHF, Germany) in the optical path and we quantified the imaging performance by measuring the variation of the pixel intensity along the seven dashed lines of a width of 2 µm, corresponding to 22 pixels. Hereby, we repositioned the line-space pattern so that the complete range 0 < *r* < 12 µm could be studied. The extracted pixel gray values were normalized, taking as hundred percent the lighter region outside of the line pattern and zero percent the darkest pixel intensity of the micro-patterned structures. The peak-to-valley distances of the thus obtained curves were measured and marked as modulation. The graphs of Fig. 4b were constructed by placing line-space patterns with 260 nm, 280 nm and 300 nm pitch, respectively, in the center of a single microsphere. Seven measurement lines were placed along the horizontal axis (shown on Fig. 4a) of the images, starting from the center with 2 µm increments. The modulation rapidly decreases as the local distance between the sample and the microsphere surface increases, in good agreement with theoretical calculations of the evanescent behavior of sub-diffraction-sized nanostructures^37^. In Fig. 4c the modulation performance of the microscope objective with (yellow) and without (purple) the presence of the microsphere array was compared by imaging line-space patterns with different pitch in the 240–400 nm range. Data analysis showed that there is a significant gain due to the use of the microsphere when the lateral dimension of the sample is below 180 nm, *i.e*. exactly in the diffraction limited region.

**Figure 5:**
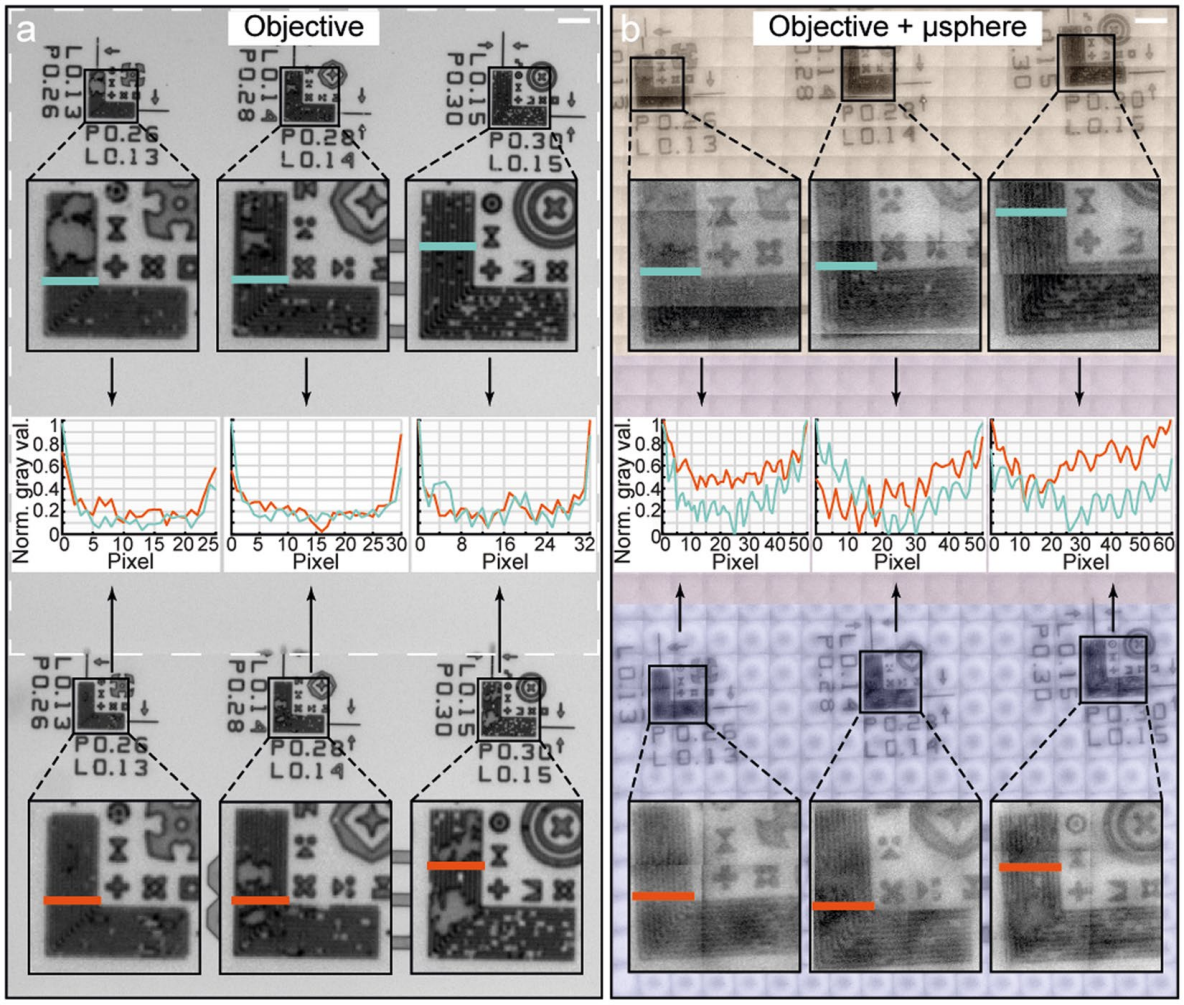
Resolution analysis of the image. (**a**) Picture of the sample taken via the microscope objective without use of the microsphere array. Since the field-of-view of the camera is smaller than the demonstrated scanned surface of the sample, two pictures were stitched together, one being marked by a white dashed rectangle. Nanostructures within the upper field-of-view are identical to the ones in the lower field-of-view, and are composed of 130 nm, 140 nm, and 150 nm L shaped line-space patterns from the left to the right, respectively. Black squares are zooms on these patterns. The optical signals are evaluated along the five pixel-wide horizontal lines (blue for the upper part and orange for the lower part) and plotted in the center as normalized gray values (a black pixel generating zero signal and a white pixel generating signal one). One can observe that the 150 nm nanostructures-generated modulation patterns are resolvable; meanwhile no modulation is observed for the 130 nm and 140 nm nanostructures. (**b**) Picture of the same sample as in (**a**) and Fig. 3c, taken via the microscope objective using the microsphere array. Yellow colored tiles were recorded by the first microsphere, and blue ones by the second microsphere. Insets show zooms on the recorded modulation patterns. Optical signals are evaluated along the same five pixel-wide lines as in (**a**). Modulation plots in the center are generated with the same method as in (**a**), showing that all nanostructures are resolvable. Scale bar 5 µm.

To benchmark the performance of our approach, we compared the composed picture to the image that was taken by the microscope camera without using a microsphere (Fig. 5). In Fig. 5a, we see the line-space patterns of row nine from the sample of Fig. 3 in the upper part, and the line-space patterns of row eight in the lower part. The white dashed rectangle shows a single field-of-view of the microscope mounted camera. To be able to make fair comparison with our composed image, we took two photographs from the microscope and stitched them together. In the insets, enlarged images of the line-space patterns are shown, clearly indicating that the microscope cannot resolve features below the diffraction limit. To further support this statement, we drew five pixel-wide measurement lines on the taken photographs (blue lines correspond to patterns of row nine, while orange lines correspond to patterns of row eight), on which we evaluated the pixel gray values. We positioned these lines on exactly the same spot for every pattern, except for the 150 nm wide lines where they are shifted up by a few microns, because of a damaged region in the pattern of row eight. To exclude the shift caused by eventual different brightness of the light source, we normalized all pixel gray values, resulting in a modulation pattern as discussed already in Fig. 4a. Therefore, on the plots in the center of Fig. 5a, the zero value corresponds to the darkest pixel and the one value corresponds to the lightest region next to the line-space pattern, *i.e*. the down-pointing peaks correspond to the dark lines in the pattern. One can observe that the peaks are distinguishable on the most right side plot (evaluating the 150 nm wide lines), but that they disappear as the line width is decreased to 140 nm (center plot) and finally to 130 nm (left side plot).

In Fig. 5b we show the image of the same area, but in this case, the picture was created with our microsphere array. We applied yellow and blue colors on the picture to show which part of it was created by the first and which by the second microsphere in our array. The insets show enlarged stitched images of the line-space patterns, with markings of the positions of our measurement lines. Just by eye observation, it is already clear that the lines, independently of their size, are more visible than in Fig. 5a. For evaluating the gray values along the measurement lines, we used the same method as described in the previous paragraph. On the plots in the center of Fig. 5b, one can observe that the peaks corresponding to the black lines on the sample are sharper and that the modulation amplitude is bigger. It is important to note, that the modulation did not change significantly between the biggest (150 nm) and the smallest (130 nm) line width, *i.e*. our imaging system could well resolve down to 130 nm wide features using a halogen light source.

**Figure 6:**
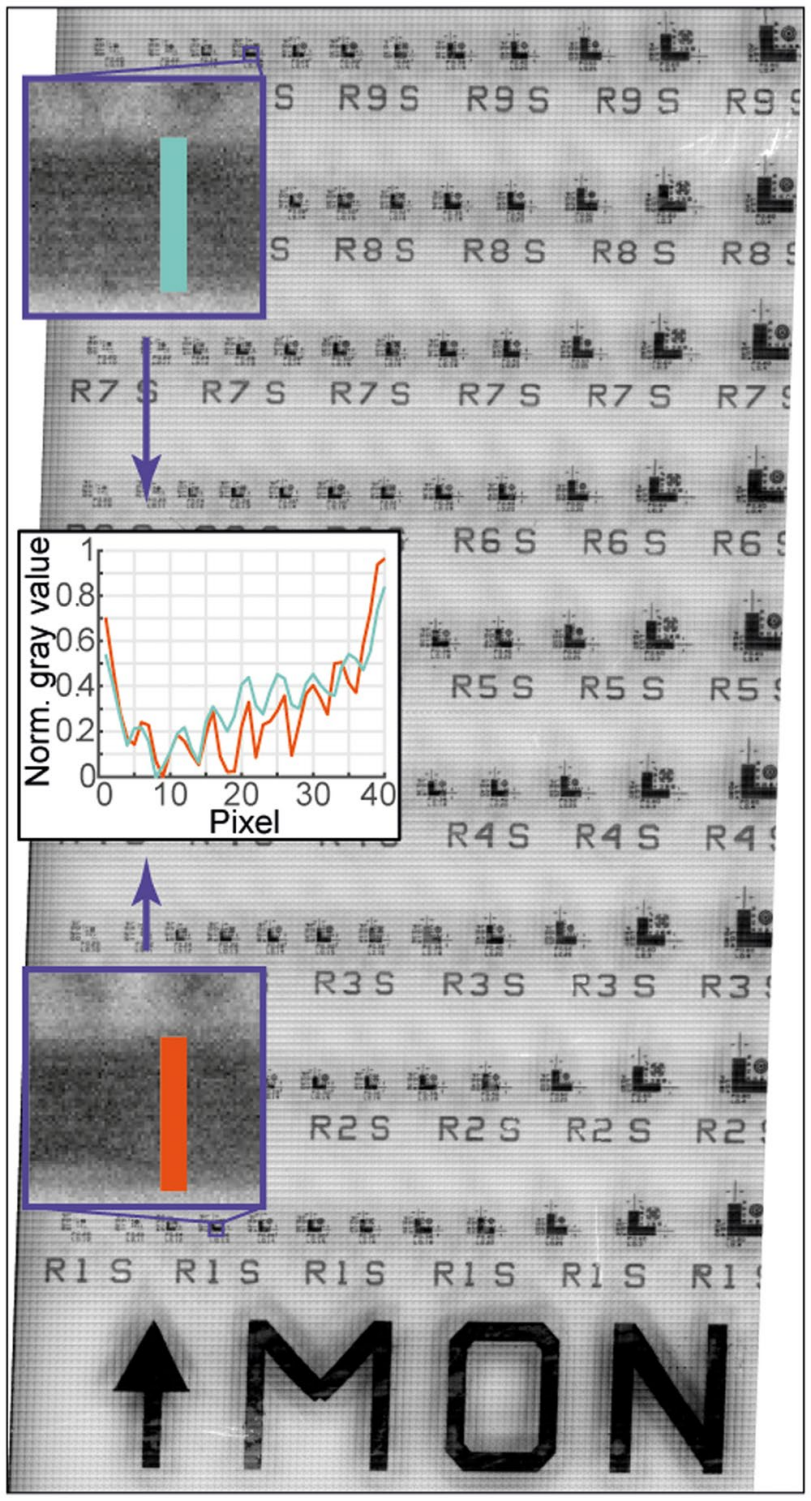
Demonstration of large area imaging with super-resolution. The sample area shown here is 0.5 mm × 1.0 mm. It was reconstructed from 20 301 individual pictures and its original file consists of ~175 MPixels. Insets show that 130 nm line-space patterns remained resolvable over the whole scanned area.

Finally, to demonstrate the robustness and full possibility of our imaging technique, we show in Fig. 6 a super-resolution imaging corresponding to a large surface area (0.5 mm × 1.0 mm). During scanning, 20 301 individual pictures were collected using our custom algorithm, resulting in ~60 GB of raw data. Our stitching algorithm composed the final image that had ~175 MPixel and ~530 MB file size. One can observe that due to the shear stress generated during the scanning, a slight systematic tilt occurred on the picture, which was corrected by our image reconstruction algorithm. The shadow effect at the edge of the tiles could not be compensated by our algorithm, therefore the quality of the stitching could be improved, *e.g*. by using seamless stitching in ImageJ, but it is important to note, that our solution completed the stitching ~100× faster than the ImageJ algorithm. As the insets in Fig. 6 show, the 130 nm lateral resolution was preserved over the total area of the scanned surface.

## Conclusion

We demonstrated an advanced implementation of an optical microscopy super-resolution imaging technique, using an ordered array of dielectric microspheres. The imaging principle was explained to be related to the existence of a photonic nanojet upon illumination of a microsphere and the near-field interactions between the sample and the microsphere. We showed that it is possible to overcome some of the field-of-view limitations of previously published microsphere-based super-resolution imaging techniques by implementing a scanning and stitching process. Our simple but smart system achieved a 240 nm pitch lateral resolution in static mode. Furthermore, 260 nm pitch and simultaneously a much bigger total field-of-view than the one of the microscope-mounted camera was demonstrated. To show the robustness of the system, a surface scan of 5 × 10^5^ µm^2^ was presented. However, we believe that even bigger areas can be imaged, since there are no intrinsic limits in our process. Later, the scanning system could eventually be optimized for mass production with the help of 3D printing, as this technique enables very flexible microfabrication of customized parts, as was shown earlier^31^. We therefore hope that our findings will help repositioning dielectric microsphere-based optical super-resolution microscopy beyond the proof-of-concept stage towards a fully operational real-life application.

### Data availability

The data that support the plots within this paper and other findings of this study are available from the corresponding author upon reasonable request.
